# Methylation-associated silencing of miR-200b facilitates human hepatocellular carcinoma progression by directly targeting *BMI1*

**DOI:** 10.18632/oncotarget.7629

**Published:** 2016-02-23

**Authors:** Wen-rui Wu, Hong Sun, Rui Zhang, Xian-huan Yu, Xiang-de Shi, Man-sheng Zhu, Hong Zeng, Li-xu Yan, Lei-bo Xu, Chao Liu

**Affiliations:** ^1^ Guangdong Provincial Key Laboratory of Malignant Tumor Epigenetics and Gene Regulation, Sun Yat-sen Memorial Hospital, Sun Yat-sen University, Guangzhou, 510120, China; ^2^ Department of Biliary-Pancreatic Surgery, Sun Yat-sen Memorial Hospital, Sun Yat-sen University, Guangzhou, 510120, China; ^3^ Department of Respiratory, The First Affiliated Hospital/School of Clinical Medical, Guangdong Pharmaceutical University, Guangzhou, 510080, China; ^4^ Department of Thoracic Surgery, Cancer Center of Guangzhou Medical University, Guangzhou, 510095, China; ^5^ Department of Pathology, Sun Yat-sen Memorial Hospital, Sun Yat-sen University, Guangzhou, 510120, China; ^6^ Department of Pathology, Guangdong General Hospital, Guangdong Academy of Medical Science, 510080, China

**Keywords:** hepatocellular carcinoma, progression, microRNA-200b, methylation, BMI1

## Abstract

This study aims to investigate the biological function of microRNA-200b and *BMI1*, predicted target of microRNA-200b in human hepatocellular carcinoma (HCC). MicroRNA-200b and *BMI1* expression in HCC tissues were evaluated by qPCR. A luciferase reporter assay was used to validate *BMI1* as a direct target of microRNA-200b. The effect of microRNA-200b on HCC progression was studied *in vitro* and *in vivo*. Methylation specific PCR (MSP) and bisulfite sequencing PCR (BSP) were used to detect the methylation status of the microRNA-200b promoter. Significant downregulation of microRNA-200b was observed in 83.3% of HCC tissues. By contrast, *BMI1* was significantly overexpressed in 66.7% of HCC tissues. The results of the luciferase assay confirmed *BMI1* as a direct target gene of microRNA-200b. Forced expression of microRNA-200b in HCC cells dramatically repressed proliferation, colony formation, cell cycle progression, and invasion. Moreover, microRNA-200b synergized with 5-fluorouracil to induce apoptosis *in vitro* and suppressed tumorigenicity *in vivo*. In addition, MSP analysis and BSP revealed that CpG sites in the promoter region of microRNA-200b were extensively methylated in HCC, with concomitant downregulation of microRNA-200b expression. Furthermore, microRNA-200b was activated in HCC cells after treatment with 5-azacytidine, whereas *BMI1* expression was clearly downregulated. Our results indicate that microRNA-200b is partially silenced by DNA hypermethylation and that it can repress tumor progression by directly targeting *BMI1* in HCC.

## INTRODUCTION

MicroRNAs (miRNAs, miRs) have critical functions in various biological processes such as development, infection, immunity, tumor development and progression [1]. Although numerous miRNAs have been associated with carcinogenesis and the progression of cancer, many of their biological roles remain to be characterized [2–4]. Members of the miR-200 family are key regulators of epithelial-mesenchymal transition (EMT), an important step in the progression of primary tumors to distant metastasis. For instance, Yeh et al. [5] reported that simultaneous silencing of miR-200c and miR-141 was likely to be responsible for the bile duct metastasis of HCC via EMT activation. However, little is known about the specific role of miR-200b in human hepatocellular carcinoma (HCC) [6, 7].

The polycomb group protein *BMI1* plays a crucial role in the regulation of cell proliferation, stem cell maintenance [8], tumorigenesis and tumor progression [9]. Overexpression of *BMI1* has been observed in several human malignancies, including HCC, and *BMI1* acts as an oncogene in some cancer types [9, 10]. We recently demonstrated that *BMI1* expression is elevated in HCC and the silencing of the *BMI1* gene inhibits the proliferation and invasiveness of human HCC cells [11, 12]. *BMI1* is also reportedly a target gene of miR-200b in tongue and prostate cancer [13, 14]. However, the exact regulatory mechanisms of *BMI1* expression and its relationship with miR-200b in the initiation and progression of HCC remain to be explored. In this study, we examined the potential roles of miR-200b and *BMI1* in the progression of HCC and explored the underlying regulatory mechanisms.

## RESULTS

### Downregulation of miR-200b in HCC is associated with *BMI1* overexpression

**Figure 1:**
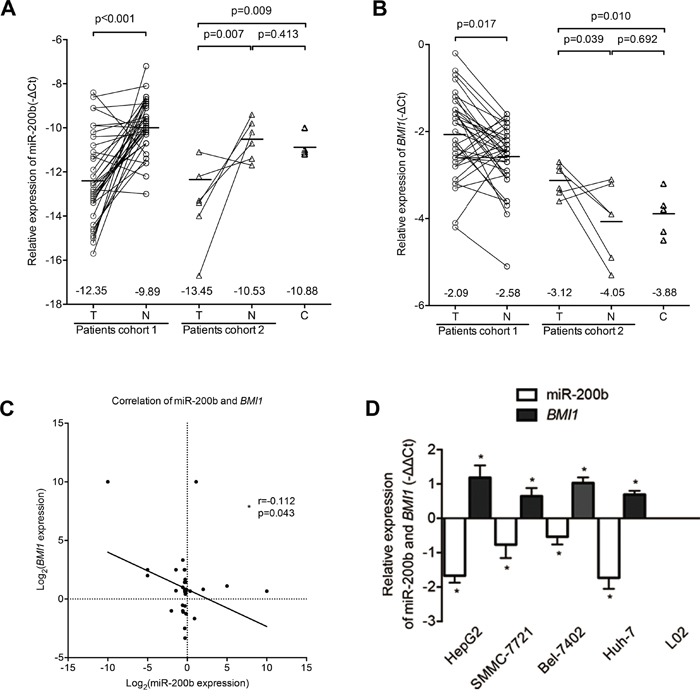
MiR-200b expression is downregulated in HCC and is associated with BMI1 overexpression **A.** miR-200b was significantly downregulated in HCC tumor tissues (T) compared with respective adjacent non-cancerous liver tissues (N) and normal liver tissues (C). **B.** *BMI1* was significantly upregulated in HCC tumor tissues. **C.** The expression of miR-200b was negatively correlated with *BMI1* expression in HCC tissues. **D.** Relative expression levels of miR-200b and *BMI1* mRNA in human liver cancer cell lines and in the normal liver cell line L02. *p<0.05

The expressions level of miR-200b and *BMI1* in HCC tissues and human liver cancer cell lines were analyzed by qPCR. MiR-200b expression was significantly decreased in 83.3% (cohort 1: 30/36; cohort 2: 5/6) of HCC tissues (Figure 1A) compared with non-cancerous tissues and in the four human liver cancer cell lines (HepG2, SMMC-7721, Bel-7402, Huh7) compared with the normal human liver cell line L02 (Figure 1D). By contrast, we detected significant upregulation of *BMI1* in 66.7% (cohort 1: 24/36; cohort 2: 4/6) of HCC tissues (Figure 1B) and in all four human liver cancer cell lines (Figure 1D). Moreover, the expression of miR-200b was negatively correlated with the expression of *BMI1* mRNA in HCC tissues in cohort 1 (Figure 1C). However, no significant correlations between miR-200b or *BMI1* expression and the available clinical parameters of patient cohort 1 were observed (Table S1).

### *BMI1* is a direct target gene of miR-200b and is negatively regulated by miR-200b in human liver cancer cell lines

**Figure 2:**
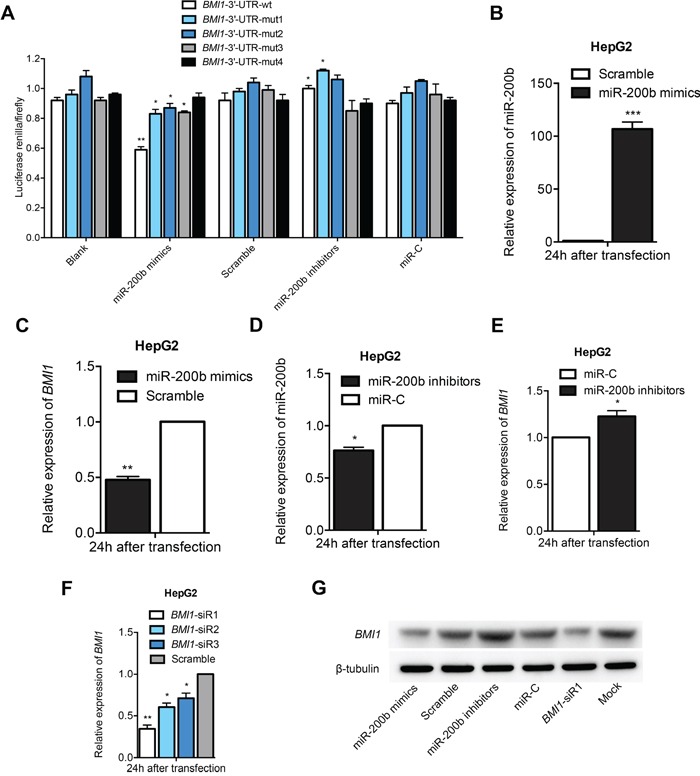
BMI1 is a direct target gene of miR-200b **A.** Luciferase assays revealed that *BMI1* is a direct target gene of miR-200b. **B, D.** Transfection of HepG2 cells with miR-200b mimics or miR-200b inhibitors specifically upregulated or downregulated miR-200b levels, respectively. **C, E, G.** Transfection of HepG2 cells with miR-200b mimics or miR-200b inhibitors significantly modulated mRNA and protein levels of *BMI1*. **F.** The knockdown effect of *BMI1*-siR1 was considerably greater than that of *BMI1*-siR2 and *BMI1*-siR3. *p<0.05, **p <0.01, ***p <0.001

To validate *BMI1* as a bona fide target gene of miR-200b, a human *BMI1* 3′-UTR fragment containing the wild type or mutant type miR-200b-binding sequence was subcloned into the *XhoI*/*NotI* site downstream of the firefly luciferase reporter gene in the psiCHECK-2 vector. Interestingly, co-transfection of the miR-200b mimics specifically decreased the luciferase expression of the *BMI1*-3′-UTR-wt reporter. By contrast, the luciferase activities of the four *BMI1*-3′-UTR-mut reporters and the psiCHECK-2 control reporter were unaffected by the simultaneous transfection of the miR-200b mimics, which suggests that *BMI1* is a direct target gene of miR-200b (Figure 2A). In addition, the results of the qPCR and western blot analysis demonstrated that transfection with miR-200b mimics significantly reduced the mRNA and protein expression levels of *BMI1* in HCC cells (Figure 2B, C and G; Figure S1). By contrast, a miR-200b inhibitor significantly enhanced the expression of *BMI1* in HCC cells (Figure 2D, E and G; Figure S1). Accordingly, the silencing of the *BMI1* gene by siRNA transfection in HCC cells resulted in the downregulation of endogenous *BMI1* mRNA and protein levels compared with the negative control (Figure 2F and G; Figure S1). Taken together, these results indicate that *BMI1* is a direct target gene of miR-200b and can be negatively regulated by miR-200b.

### MiR-200b suppresses proliferation, colony formation, cell cycle progression and invasion *in vitro*

**Figure 3:**
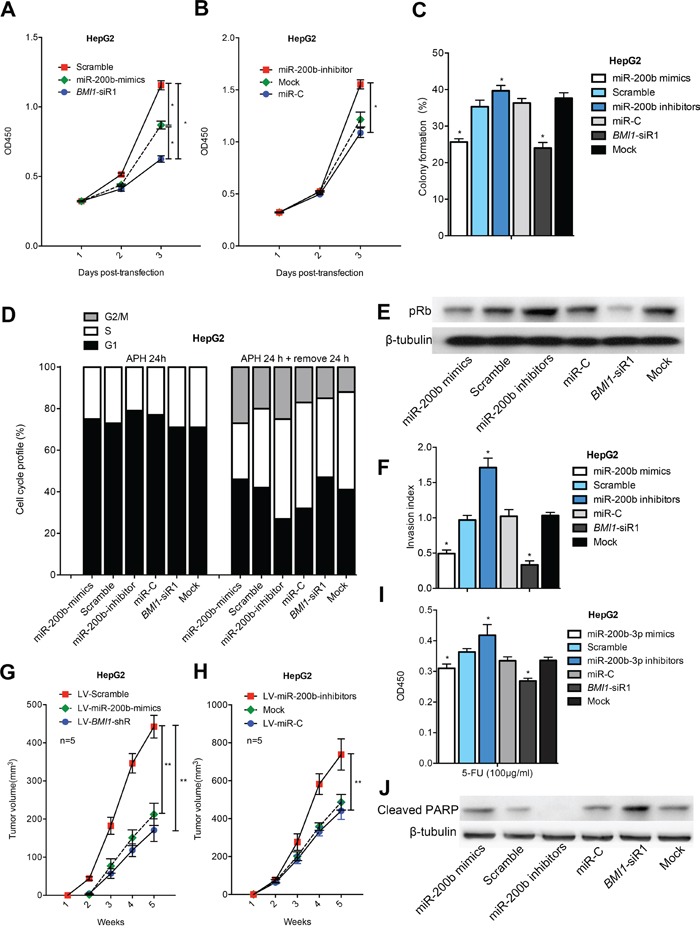
MiR-200b represses the growth and invasion of HCC cells and sensitizes HCC cells to apoptosis MiR-200b mimics/*BMI1*-siRNA repressed the **A, B.** proliferation and **C.** colony formation ability of HepG2 cells. **D.** miR-200b retarded G1/S cell cycle transition. **E.** MiR-200b mimics/*BMI1*-siRNA efficiently inhibited the expression of pRb. **F.** Either miR-200b overexpression or *BMI1* knockdown repressed the invasive capacity of HepG2 cells. **G, H.** Transfection of LV-miR-200b mimics/LV-*BMI1*-shRNA suppressed tumor formation in a nude mouse xenograft model. **I, J.** MiR-200b mimics/*BMI1*-siRNA reduced cell viability **I.** and increased the level of Cleaved PARP **J.** in 5-FU-treated HCC cells. *p <0.05, **p <0.01

The significant downregulation of miR-200b expression in HCC samples and cell linesprompted us to investigate the biological significance of miR-200b in hepatocarcinogenesis. We found that the expression of miR-200b mimics and *BMI1*-siRNA significantly decreased the proliferation capacity of HCC cells (Figure 3A and B; Figure S2). As shown in Figure 3C, miR-200b-transfected cells and *BMI1*-siRNA-transfected cells displayed much fewer and smaller colonies than control-transfected and non-transfected cells. By contrast, knockdown of miR-200b by miR-200b inhibitors significantly increased the number and size of colonies compared with the controls (Figure 3C; Figures S3 and S4). These results indicate that overexpression of miR-200b in HCC cells may contribute to decreased cell proliferation and colony formation via the functional downregulation of *BMI1* expression.

Next, cell cycle distribution analysis was performed to investigate whether the effects of miR-200b and *BMI1* are mediated by cell cycle regulation. Upon treatment with aphidicolin (5 μM), more than 70% of the cells underwent growth arrest at the G0/G1 stage, and no difference was observed among the six groups in terms of cell cycle distribution. After removal of aphidicolin, transfection of miR-200b mimics or *BMI1* siRNA triggered significant growth arrest of HCC cells at G1 phase, suggesting that the G1/S cell cycle transition is slowed by miR-200b-mediated *BMI1* silencing (Figure 3D; Figures S5 and S6). One function of the retinoblastoma protein (Rb) is to prevent excessive cell growth by preventing the progression from G1 to S phase of the cell cycle [15]. We observed that miR-200b mimics or *BMI1*-siRNAtransfection induced a higher rate of Rb dephosphorylation compared with the controls, consistent with the accumulation of cells in G1 phase (Figure 3E; Figure S6).

We previously demonstrated that knockdown of *BMI1* inhibits the invasive properties of human HCC cells [11]. Because miR-200b is a negative regulator of *BMI1*, we performed an *in vitro* cell invasion assay to further determine if miR-200b affects cell invasion. As expected, transfection of HCC cells with miR-200b mimics or *BMI1*-siRNA notably suppressed their invasion ability compared with normal control cells (Figure 3F; Figures S7 and S8). Collectively, the above results suggest that miR-200b not only suppresses growth but also inhibits the invasiveness of tumor cells by directly targeting *BMI1*.

### Exogenous overexpression of miR-200b suppresses tumorigenicity *in vivo*

The *in vitro* findings were confirmed using animal models. Nude mice were injected subcutaneously in opposite flanks with control lentiviral vector-infected cells and miR-200b mimics/*BMI1*-shRNA vector-infected cells. Consistent with the results of our *in vitro* study, enhanced expression of miR-200b or decreased expression of *BMI1* in HCC cells significantly suppressed their tumorigenicity *in vivo* (Figure 3G; Figures S9 and S10). The above findings were supported by loss-of-function studies in which a miR-200b inhibitor that significantly decreased endogenous miR-200b expression promoted *in vivo* tumorigenicity of HCC cells (Figure 3H; Figures S9 and S10). Taken together, these data indicate that either the transfection of HCC cells with miR-200b or the knockdown of *BMI1* in HCC cells significantly inhibits tumorigenicity *in vivo*.

### MiR-200b sensitizes HCC cells to apoptosis

To determine if miR-200b overexpression sensitizes HCC cells to chemotherapy, we evaluated the effect of miR-200b overexpression on cell apoptosis using an apoptosis assay. Importantly, miR-200b overexpression or *BMI1* knockdown caused a significant decrease in the viability of HCC cells after 5-fluorouraci (5-FU) treatment compared with the respective controls. By contrast, the downregulation of miR-200b in HCC cells by a miR-200b inhibitor significantly increased the cell viability of these cells (Figure 3I; Figure S11). Western blot analysis demonstrated that the levels of an apoptotic marker (Cleaved PARP) were significantly elevated in miR-200b mimic/*BMI1*-siRNA-transfected cells, whereas pre-treatment with a miR-200b inhibitor significantly attenuated the levels of Cleaved PARP (Figure 3J; Figure S11). The above findings indicate that miR-200b sensitizes HCC cells to chemotherapeutic drug-induced apoptosis via targeting of *BMI1*.

### Hypermethylation of the miR-200b gene promoter in HCC

**Figure 4:**
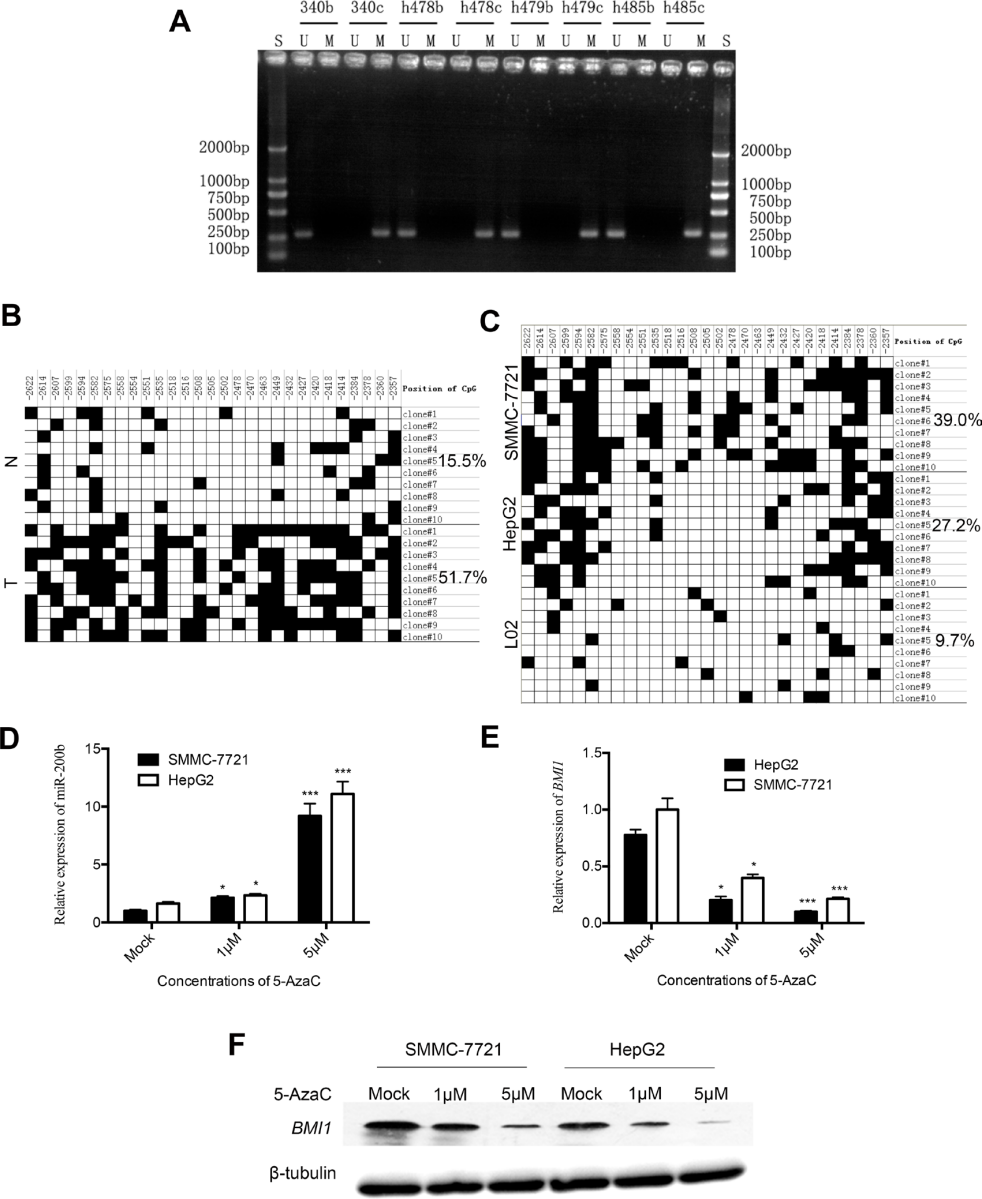
Analysis of miR-200b methylation status in HCC tissues and cell lines **A.** Results of the MSP analysis of miR-200b in HCC tissues. **B.** BSP in selected HCC tumor (T) and non-cancerous liver tissues (N) (case h479). The filled boxes denote methylated CpGs. **C.** BSP in liver cancer cell lines (HepG2, SMMC-7721) and in the normal liver cell line L02. **D.** miR-200b expression was upregulated in HepG2 and SMMC-7721 cells upon treatment with 5-AzaC for 36h. **E, F.** mRNA and protein levels of *BMI1* were downregulated upon treatment with 5-AzaC for 36h.

To investigate the mechanisms underlying the aberrant expression of miR-200b in HCC, we analyzed the promoter methylationstatus of miR-200b by MSP. As shown in Figure 4A, all cancerous samples exhibited miR-200b promoter methylation, whereas no methylation was detected in adjacent non-cancerous liver tissues. Subsequently, BSP was used to analyze the methylation status of all the CpG sites of the miR-200b promoter to precisely quantify the degree of methylation of the miR-200b promoter in one HCC sample and in two HCC cell lines. As illustrated in Figure 4B, a significantly higher level of DNA methylation was detected in HCC tumor tissue compared with respective adjacent non-cancerous liver tissue. These data are consistent with the MSP methylation results. In addition, BSP revealed high levels of DNA methylation in HepG2 and SMMC-7721 cells, consistent with the downregulation of the expression of miR-200b in these cell lines. By contrast, only limited methylation was observed in the normal liver cell line L02 (Figure 4C), which highly express miR-200b. Collectively, these results suggest that miR-200b expression is inversely correlated with DNA methylation.

### MiR-200b is activated in HCC cells after treatment with 5-AzaCand associated with silencing of *BMI1*

To further determine if miR-200b is silenced through epigenetic mechanisms in HCC cells, we compared the expression of miR-200b in HepG2 and SMMC-7721 cells treated with 5-AzaC (a DNA hypomethylating agent) to that in untreated cells by qPCR. The treatment with 5-AzaC increased the expression of miR-200b in both cell lines in a dose-dependent manner (Figure 4D). In general, the expression of miR-200b targets should decrease in cells after treatment with DNA hypomethylating drugs. To confirm this relationship, we monitored the expression levels of *BMI1* in extracts of 5-AzaC-treated cells by qPCR and western blot analysis. As expected, the expression of *BMI1* was clearly downregulated in HCC cells upon treatment with 5-AzaC in a dose-dependent manner (Figure 4E and F). Taken together, these results indicate that miR-200b expression in HCC may be epigenetically downregulated via DNA methylation and that *BMI1* was a functional downstream target of miR-200b.

## DISCUSSION

We previously determined that miR-101 can inhibit the progression of HCC via downregulation of enhancer of zeste homolog 2 (EZH2; polycomb group protein) [2]. In this study, we demonstrated that the tumor suppressor miR-200b represses HCC progression by directly targeting the oncogene *BMI1*. Furthermore, our data indicate that miR-200b is partially silenced by DNA hypermethylation.

Accumulating evidence suggests that miRNAs regulate a variety of physiological functions via the regulation of their target gene-related signaling pathways [16]. The previously identified tumor suppressor miR-200b modulates tumor development and malignant progression by downregulating its target genes. Sun and colleagues [13] have demonstrated that miR-200b can inhibit chemotherapy-induced EMT in human tongue cancer cells by targeting *BMI1*. In this study, we observed that the expression of miR-200b was downregulated in most of the HCC tissues tested and that the miR-200b level was inversely correlated with the expression level of *BMI1*. Luciferase assays revealed direct inhibition of *BMI1*-3′-UTR expression by miR-200b. In agreement with these data, qPCR and western blot analysis demonstrated that miR-200b could inhibit the mRNA and protein expression of *BMI1* in HCC cells. Taken together, these results strongly suggest that *BMI1* is a direct target gene of miR-200b in HCC, thus indicating the underlying mechanism by which miR-200b regulates *BMI1* at both the transcriptional and post-translational levels.

Emerging evidence suggests that EMT can promote cancer cell motility and plasticity and can fuel both tumor initiation and metastatic spread [17]. The miR-200 family plays a vital role in tumor suppression through the inhibition of EMT [6, 7]. MiR-200b can restrain the process of EMT and consequently inhibit EMT-mediated tumor invasion and metastasis. MiR-200b is therefore considered an important oncomir in many types of cancer [18–20]. Here, we demonstrated that miR-200b not only suppresses invasion but also inhibits the growth of HCC cells via the direct targeting of *BMI1*. Ectopic overexpression of miR-200b also dramatically inhibited HCC cell proliferation and colony formation, thereby contributing to the delay in HCC progression. These effects may be mediated by the inhibition of the *BMI1* oncogene, which would lead to a higher rate of Rb dephosphorylation and the accumulation of cells in G1 phase. In addition, tumor growth inhibition was also observed *in vivo* in HepG2 and SMMC-7721 xenografts.

An increasing number of miRNAs have been linked to chemotherapeutic drug induced apoptosis [21]. We previously demonstrated that forced expression of miR-101 can sensitize HCC cells to chemotherapeutic treatment by directly targeting the *EZH2* oncogene [2]. In the present study, miR-200b-mediated *BMI1* silencing sensitized HCC cells to 5-FU-induced apoptosis, whereas an inhibitor of miR-200b antagonized the pro-apoptotic effect, suggesting that miR-200b may increase chemotherapeutic drug-induced cytotoxicity.

The crucial role of miR-200b and its target gene *BMI1* in the progression of HCC prompted us to further explore the mechanisms underlying the aberrant expression of miR-200b in HCC. Recent studies have demonstrated that DNA hypermethylation of CpG islands within gene promoter can downregulate tumor suppressor genes in human cancers [22]. He et al. [23] reported that DNA methylation plays an important and complex role in the regulation of miRNA expression in HCC. Our study revealed that hypermethylation of the CpG islands upstream of miR-200b led to the downregulation of miR-200b in 2 HCC cell lines and tissues from 4 HCC patients. Moreover, treatment with the DNA hypomethylating agent 5-AzaC increased the expression of miR-200b in HCC cell lines, whereas the expression of its target gene *BMI1* was upregulated. These findings indicate that the downregulation of miR-200b in HCC is due, at least in part, to the hypermethylation of CpG sequences in its promoter.

In summary, our results indicate that miR-200b is partially silenced by DNA hypermethylation and can repress tumor progression by directly targeting *BMI1* in HCC. Our study suggests that miR-200b plays an important role in the progression of HCC and may be a potential therapeutic target in cancer progression.

## MATERIALS AND METHODS

### Patients and tissue samples

Tissue samples were obtained from patients with HCC (n = 42) and benign tumors (n = 6) in the Department of Biliary-Pancreatic Surgery, SunYat-Sen Memorial Hospital, Sun Yat-Sen University (Guangzhou, People's Republic of China) from January 2000 to December 2011. Pathologic diagnosis was confirmed by two pathologists independently. All specimens obtained within 20 minutes after resection and were immediately frozen in liquid nitrogen until further use. Informed consent was obtained from all subjects according to the Internal Review and Ethics Boards of SunYat-Sen Memorial Hospital, and the project was in accordance with the Helsinki Declaration of 1975. Clinicopathologic characteristics are presented in Tables S1 and S2.

### Cell culture

Human HCC cell lines HepG2, SMMC-7721, Bel-7402, Huh7, and normal liver cell line L02 were obtained from the Type Culture Collection of the Chinese Academy of Sciences (Shanghai, China). SMMC-7721 and BEL-7402 cell lines were cultured in RPMI 1640 medium (Gibco, Life Technologies Corporation, Carlsbad, CA, USA) supplemented with 10% fetal bovine serum (Biological Industries, Kibbuiz, Israel). HepG2, Huh7 and L02 were cultured in DMEM medium (Gibco) supplemented with 10% fetal bovine serum (Biological Industries). All cells were maintained at 37°C in a humidified incubator containing 5% CO_2_.

### The bioinformatics of miR-200b

The predicted targets of miR-200b and their target sites were analyzed usingmiRanda (http://www.microrna.org/microrna/home.do), miRBase (http://www.mirbase.org/) and TargetScan (http://www.targetscan.org/). We search and obtain the 2kb DNA gene sequence of 5′-upstream of miR-200b by bioinformatics genome database UCSC (http://genome.ucsc.edu/) and one CpG island were found (Criteria used: Island size > 100 bp, GC Percent > 50.0%, Obs/Exp > 0.6). We carry out promoter search by means of ITFP (http://itfp.biosino.org/itfp/TFSearcher/index.jsp).

### RNA preparation and quantitative real-time PCR (qPCR) assay

RNA was extracted and qPCRwas conducted as already described [12]. The sequences of all primers are provided in Table S3.

### Western blot analysis

Western blot analysis was performed as described in our previous study [12], with the following primary antibodies: anti-*BMI1* (1:400; Abcam, Cambridge, MA, USA), anti-phosphorylated retinoblastoma (pRb; 1:1000; Cell Signaling Technology, Damvers, MA, USA), anti-Cleaved PARP (poly adenosine diphosphate ribose polymerase; 1:500; Cell Signaling Technology) or anti-β-tubulin (1:2000; Cell Signaling Technology).

### Transfection

All miRNA mimics, inhibitors and *BMI1* siRNAs were obtained from GenePharma (Shanghai, China) and their sequences were shown in Table S4. Lipofectamine 2000 (Invitrogen, Carlsbad, CA, USA) was used as a transfection reagent, and transfections were performed according to manufacturers' recommendations (described in details elsewhere [12]).

### Vector construction and luciferase reporter assay

The region of human *BMI1*-3′-untranslated region (UTR; bases 8334 to 10276) containing three putative miR-200b-binding sites (miRanda; Figure S12). A wild type (wt) and four mutant type (mut) 3′-UTR segments of human *BMI1* mRNA were amplified and subcloned into *XhoI* and *NotI* restriction sites downstream of the luciferase reporter gene in the psiCHECK-2 vector (Applied Biosystems, Foster, CA, USA). Primer sequences for amplification of *BMI1*-3′-UTR are listed in Table S5 (*BMI1*-3′-UTR-mut4: three putative miR-200b-binding sites are all mutated by double mutation). All clones were subjected to sequencing to verify the correctness of the nucleotide sequences. Luciferase activities were assayed using a Dual-Luciferase Reporter Assay system (Promega, Madison, WI, USA) according to the manufacturer's instructions.

### Generation of stably-transfected HCC cell lines for constitutive miR-200b and *BMI1*-shRNA expression

Human miR-200b mimics/inhibitors and *BMI1*-shRNA oligoswere designed and cloned into the pGLV3/H1/GFP+Purolentiviral vector (between the *BamHI* and *EcoRI* site). Oligos and primer sequences for generation of Lentiviral-miR-200b/*BMI1*-shRNA are listed in Table S6. HepG2 and SMMC-7721 cells were infected with our newly recombinant lentiviruses and negative control. Virus-containing media were changed with fresh culture medium after 12 - 24 h of infection. All cells were exchanged with fresh medium containing puromycin to select for stably transfected cells. The transduction efficiency and stability of the transfectants were monitored by FACS analysis (Figure S13). The *BMI1* protein expressions in these cells were checked by western blot analysis.

### Cell viability, colony formation, cell cycle, cell invasion, apoptosis analyses and tumorigenicity assays in nude mice

Cell viability, colony formation, cell cycle, cell invasion, apoptosis analyses and tumorigenicity assays in nude mice were performed as described in the Supplementary Materials and Methods.

### DNA extraction, methylation-specific PCR (MSP) and bisulfite sequencing PCR (BSP)

Genomic DNA from patient samples and cell lines was isolated with Easypure Genomic DNA kit (TransGen Biotech, Beijing, China). MSP was conducted by simultaneous use of primers for methylated and unmethylated forms (Table S3). The BSP primer (Table S3) was designed by Methprimer. Amplified PCR Products were purified and cloned into pMD19-T (TaKaRa). Ten clones of the selected pair of tumor and adjacent non-tumor tissue samples and each cell were sequenced, respectively. Percentage of methylation was calculated by QUMA (http://quma.cdb.riken.jp/top/index.html) [24].

### 5-Azacytidine (5-AzaC) demethylation treatment

For demethylation studies, HepG2 and SMMC-7721 cells were treated with the indicated concentrations of 5-AzaC (Sigma-Aldrich, St. Louis., MO, USA) for 36 h. The cells were then harvested for qPCR and western blot analysis.

### Statistical analysis

All statistical analysis was performed using SPSS for Windows (version 17.0, SPSS, Chicago, IL, USA). All experiments for cell cultures were carried out independently at least three times and in triplicate each time. All data were expressed as mean ± standard deviation (SD) unless otherwise indicated. We determined the significance of differences in the human HCC data using χ2 test and Pearson's correlation test, in the *in vitro* data using Student's t test, and in the *in vivo* data using the Mann-Whitney U test. In all cases, p values < 0.05 were considered statistically significant.

Supplementary Information accompanies the paper on the *Oncotarget* website.

## SUPPLEMENTARY DATA FIGURES AND TABLES


