# Supplementary material for: Methylation-associated silencing of miR-200b facilitates human hepatocellular carcinoma progression by directly targeting BMI1
Source: Oncotarget. 2016 Feb 23;7(14):18684–93. doi: 10.18632/oncotarget.7629 (PMC4951320; doi:10.18632/oncotarget.7629)
Supplement: Supplementary file 1 [file oncotarget-07-18684-s001.pdf]

SUPPLEMENTARY DATA

Cell proliferation analysis

Cell proliferation was determined by the Cell Counting Kit-8 (CCK-8, Dojindo, Tokyo, Japan) according to the manufacturer's protocol. Briefly, transfected cells (5×10^3) on a 96-well plate with 5 replicate wells were allowed to incubate for different time interval from day 1 to 3. After washed in PBS, 100 µl medium containing 10% CCK-8 solution was added to each well and incubated for 2 h at 37 °C. Samples are read directly in the wells using an absorbance of the 450 nm wavelength by an enzyme linked immunosorbent assay (ELISA) plate reader. The blank control absorbance (100 µl medium containing 10% CCK-8 alone) was subtracted from the experimental absorbance to adjust the background.

Colony formation analysis

Colony formation assays were used to estimate the clonogenicity of HCC cells that had received different treatments. 24 h after transfection, transfected cells were seeded in 6-well tissue culture plates (500 cells per well). After an incubation period of 10 days, the medium was decanted and each well was washed twice with PBS. The cells were fixed in 100% methanol for 20 min and stained with 1% crystal violet for 15 min, followed by detaining. The numbers of colonies (>20 cells/colony) were counted and pictures were captured.

Cell cycle analysis

For cell cycle analysis, cells were transfected as indicated before and treated with or without 5 µg/mL of aphidicolin (Sigma-Aldrich, St. Louis., MO, USA) for additional 24 h. After fixation, cells were incubated in Dulbecco's Phosphate- Buffered Saline (DPBS) containing 20 µg/mL of propidium iodide (Sigma-Aldrich), 200 µg/ml of RNase A, and 0.1% Triton X-100, and analyzed for cell cycle distribution by flow cytometry (FACSCalibur flow cytometer; BD Biosciences, Franklin Lakes, NJ, USA).

Cell invasion assay

Cell invasion assays were conducted using BD BioCoat Matrigel Invasion chambers (BD Biosciences, MA, USA). Briefly, 24 h after transfection, 1×10^5 cells in 0.1 ml serum-free medium were placed into the upper chamber (24-well plate) of the insert with Matrigel, whereas the lower chamber was filled with 0.6 ml conditioned medium containing 10% FBS. After 24 h of incubation, cells that were still on the upper side of the filters were mechanically removed with cotton swabs. Cells that migrated to the lower side were fixed with 4% paraformaldehyde for 20 min and were counterstained with 0.1% crystal violet for 15 min. The cells that had migrated into the lower chamber were observed and counted under a light microscope. Finally, the number of the migrating cells was calculated.

Apoptosis analyses

Twenty-four hours after transfection, cells were treated 5-fluorouracil (5-FU; Sigma-Aldrich) for 48 h. The cell viability was determined by CCK-8 assay as described in "cell proliferation assay".

In vivo xenograft study

All animal experimentation described in this study was performed in accordance with protocols approved by the Institutional Animal Care and Use Committee at Sun Yat-sen University. Briefly, 3×10^6 cells were suspended in 100 µl PBS and injected subcutaneously into 6 groups of animals each consisting of 5 female BALB/c athymicmude mice aged 4 to 6 weeks (wk). Mice were monitored for tumor growth every other day. Tumor Length (L) and width (W) were measured with a caliper and calculated using the formula of $W^2 \times L \times 0.5$.

SUPPLEMENTARY FIGURES AND TABLES

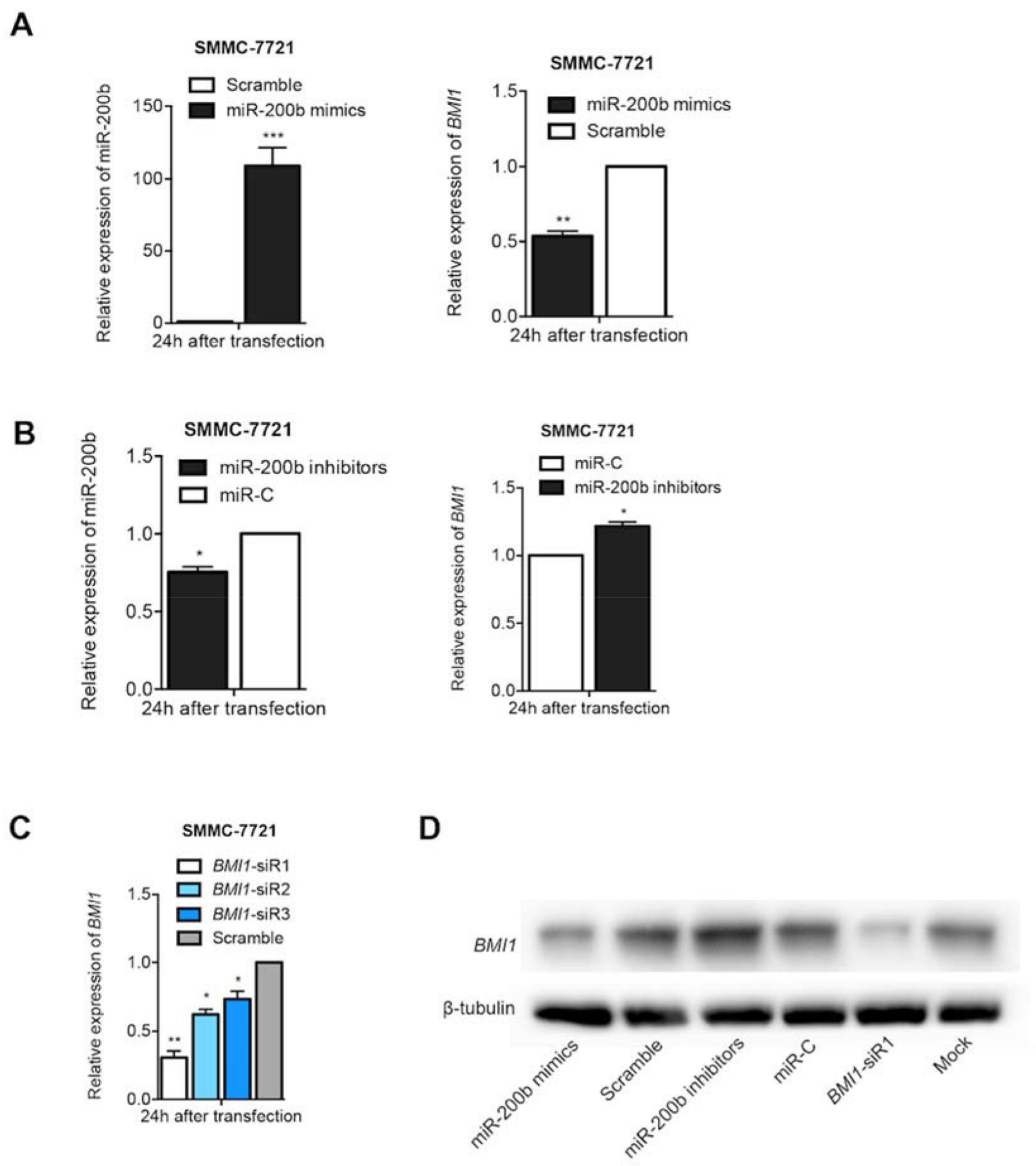

Figure S1: *BMI1* can be negatively regulated by miR-200b in SMMC-7721 cells. qPCR and western blot results revealed that transfection with miR-200b mimics, inhibitors and *BMI1*-siRNA significantly modulated mRNA **(A, B, C)** and protein **(D)** levels of *BMI1*. *p<0.05, ** p <0.01, ***p <0.001

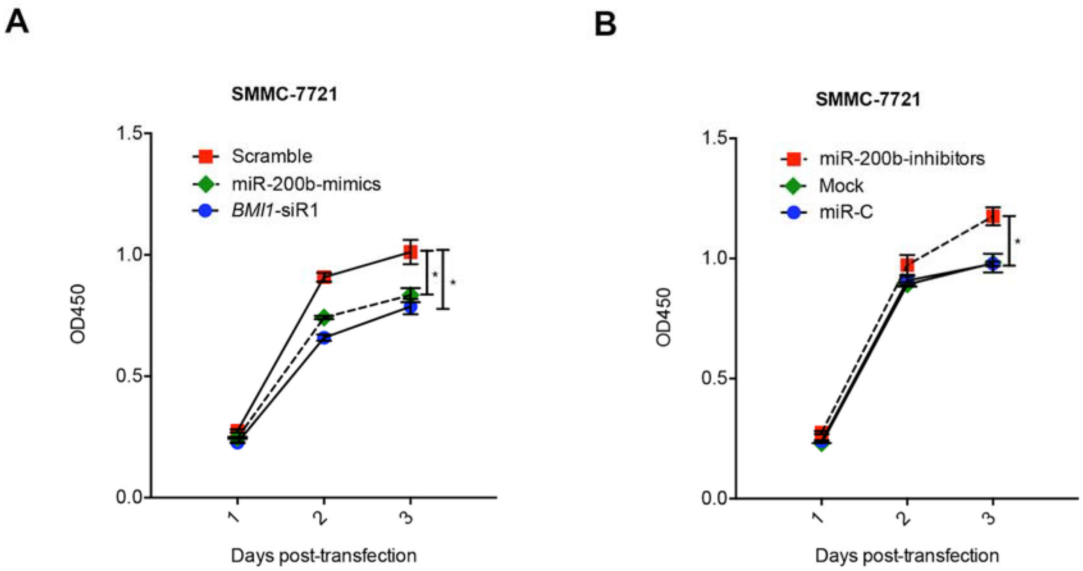

Figure S2: Transfection of miR-200b mimics or knockdown of BMI1 represses proliferation of SMMC-7721 cells *in vitro*. *p<0.05

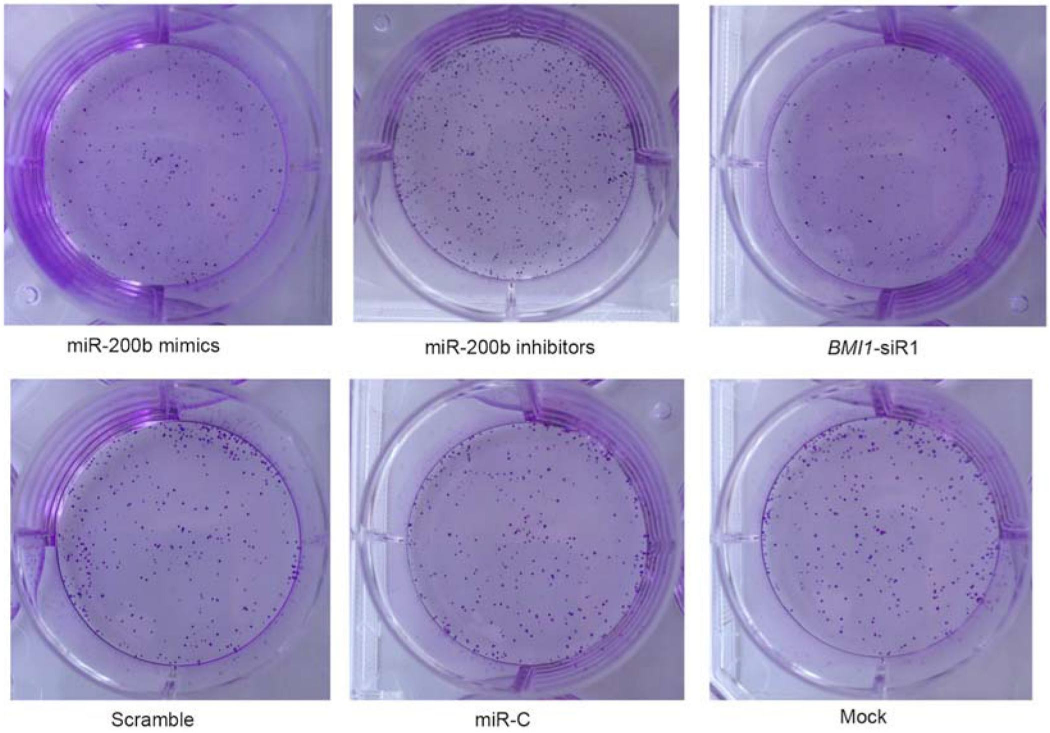

Figure S3: Representative results of colony formation of HepG2 cells after transfection of miR-200b mimics or *BMI1*-siRNA.

A

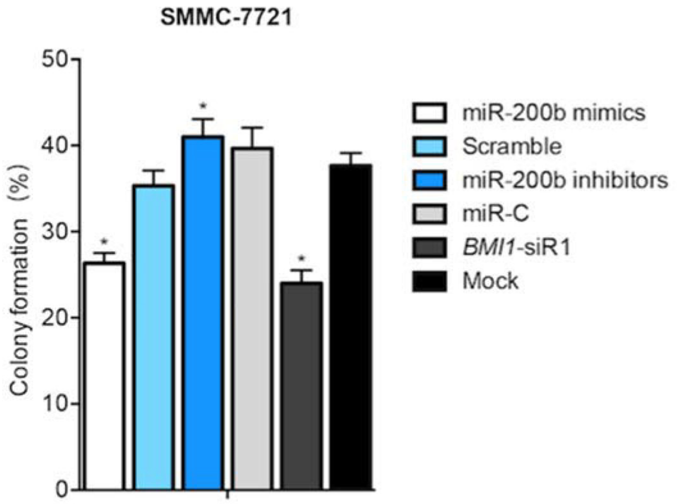

B

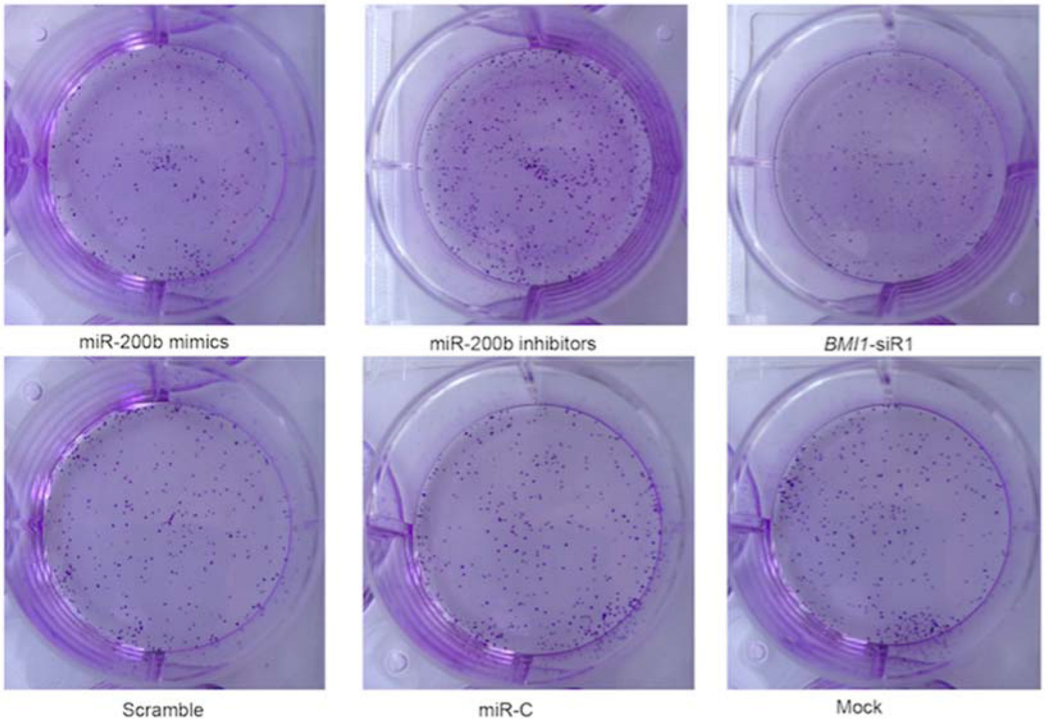

Figure S4: Transfection of miR-200b mimics or knockdown of BMI1 represses colony formation of SMMC-7721 cells *in vitro*. A. Transfection of miR-200b mimics or knockdown of *BMI1* repressed colony formation of SMMC-7721 cells. **B.** Representative results of colony formation of SMMC-7721 cells after transfection of miR-200b mimics or *BMI1*-siRNA. *p<0.05

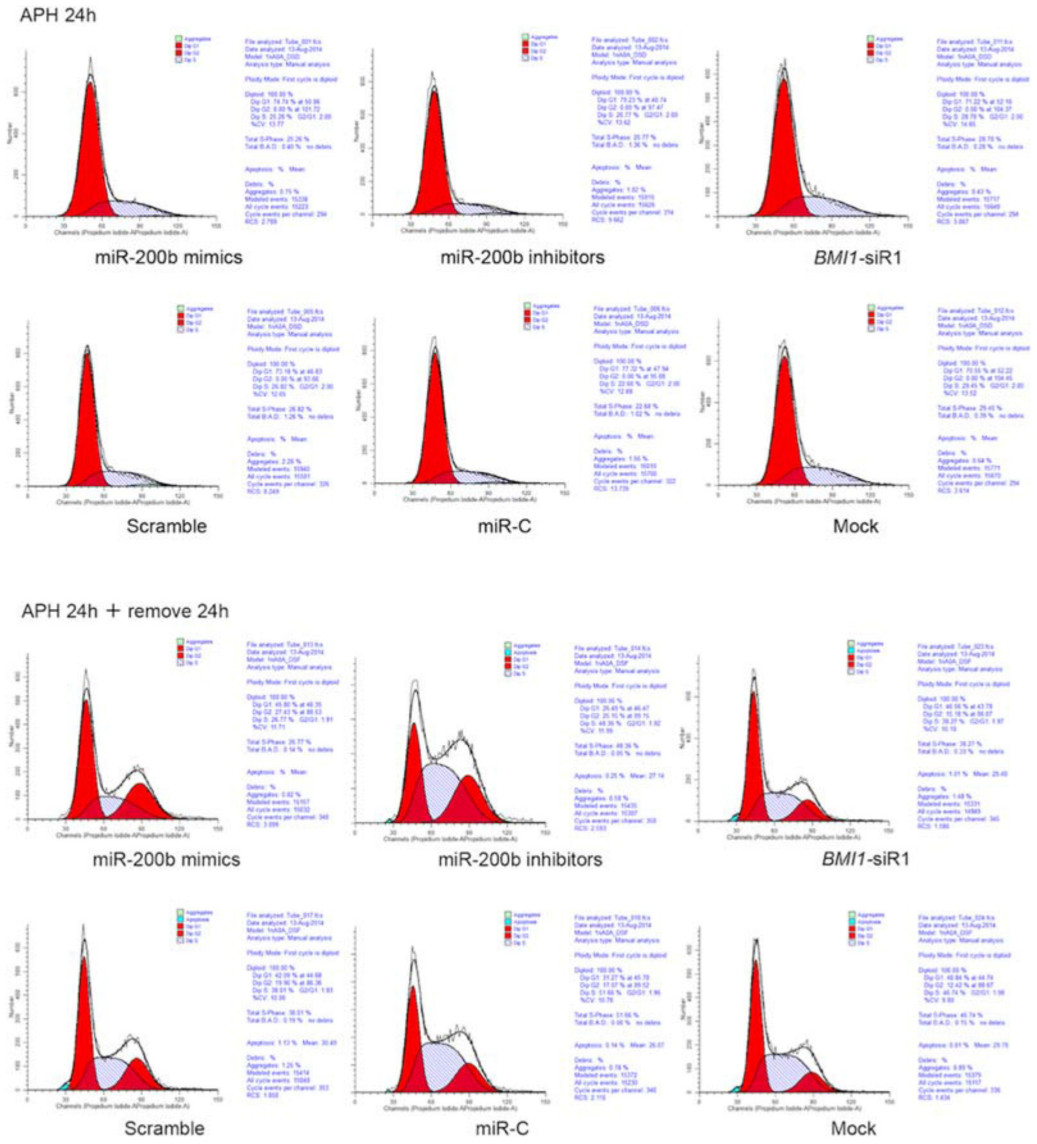

Figure S5: Representative results of cell cycle distribution of HepG2 cells after transfection of miR-200b mimics or *BMI1*-siRNA.

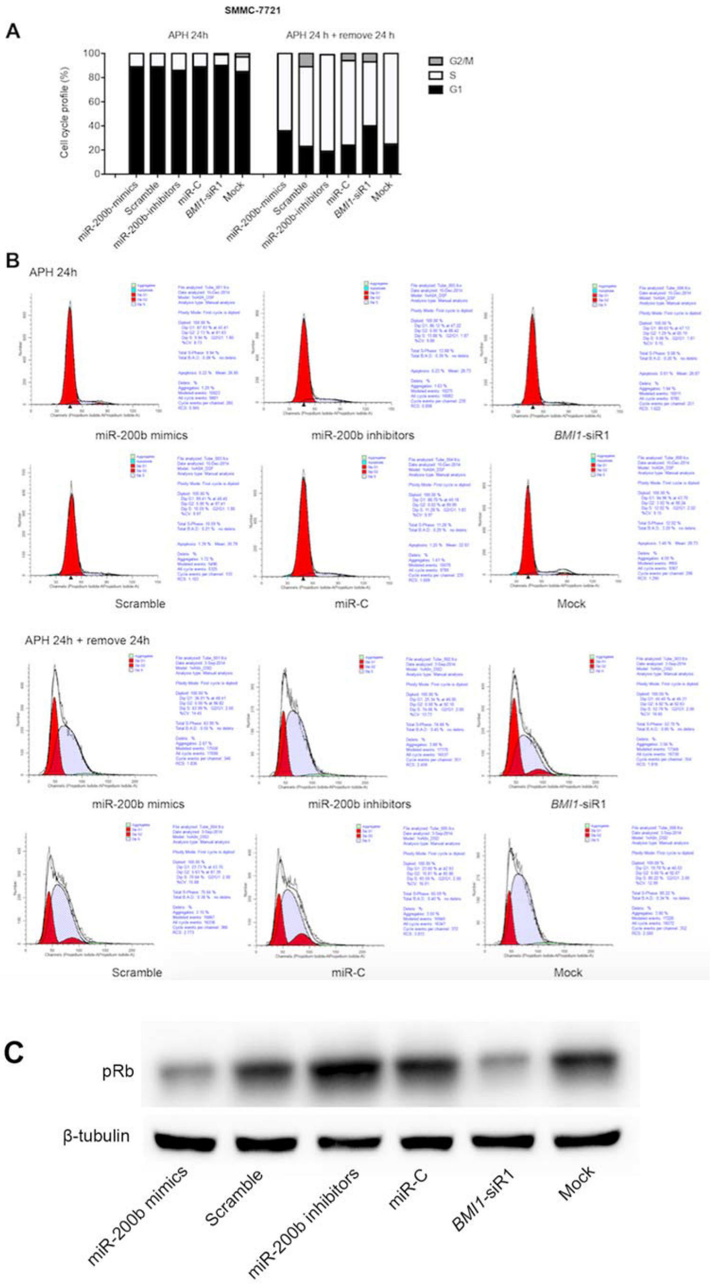

Figure S6: Transfection of miR-200b mimics or knockdown of BMI1 blocks cell cycle progression of SMMC-7721 cells *in vitro*. **A.** Transfection of miR-200b mimics or knockdown of *BMI1* blocked the cell cycle progression of SMMC-7721 cells. **B.** Representative results of cell cycle distribution of SMMC-7721 cells after transfection of miR-200b mimics or *BMI1*-siRNA. **C.** Dephosphorylation of Rb under G1 cell cycle arrest was mediated by miR-200b mimics/*BMI1*-siRNA transfection.

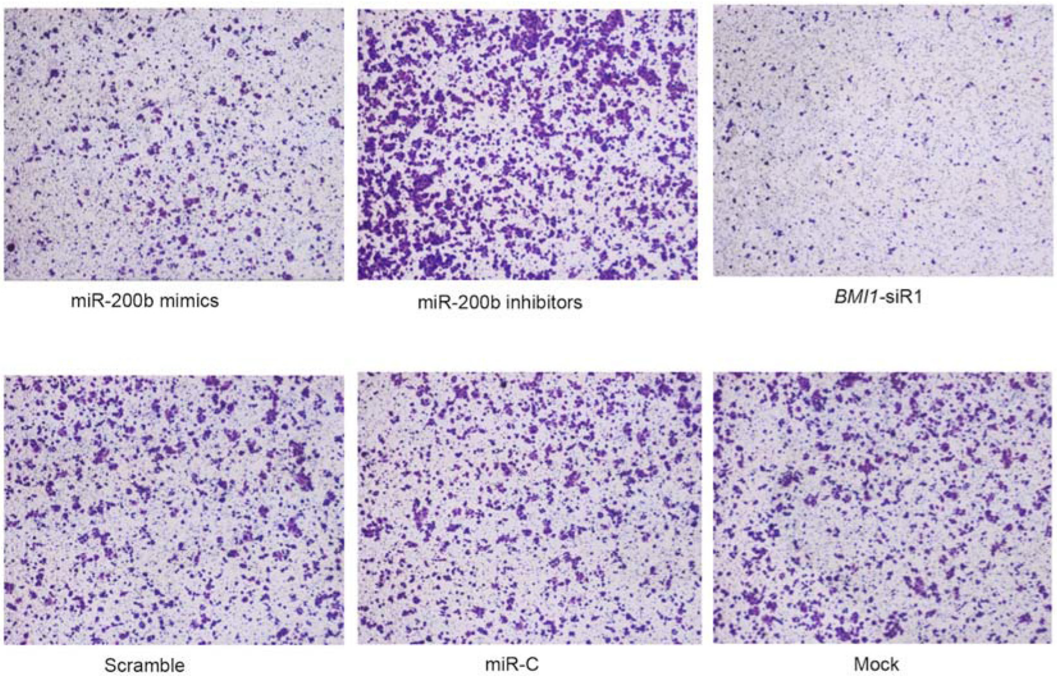

Figure S7: Representative results of miR-200b transfected or *BMI1* silenced HepG2 cells that crossed the collagen-coated membranes within the invasion chambers.

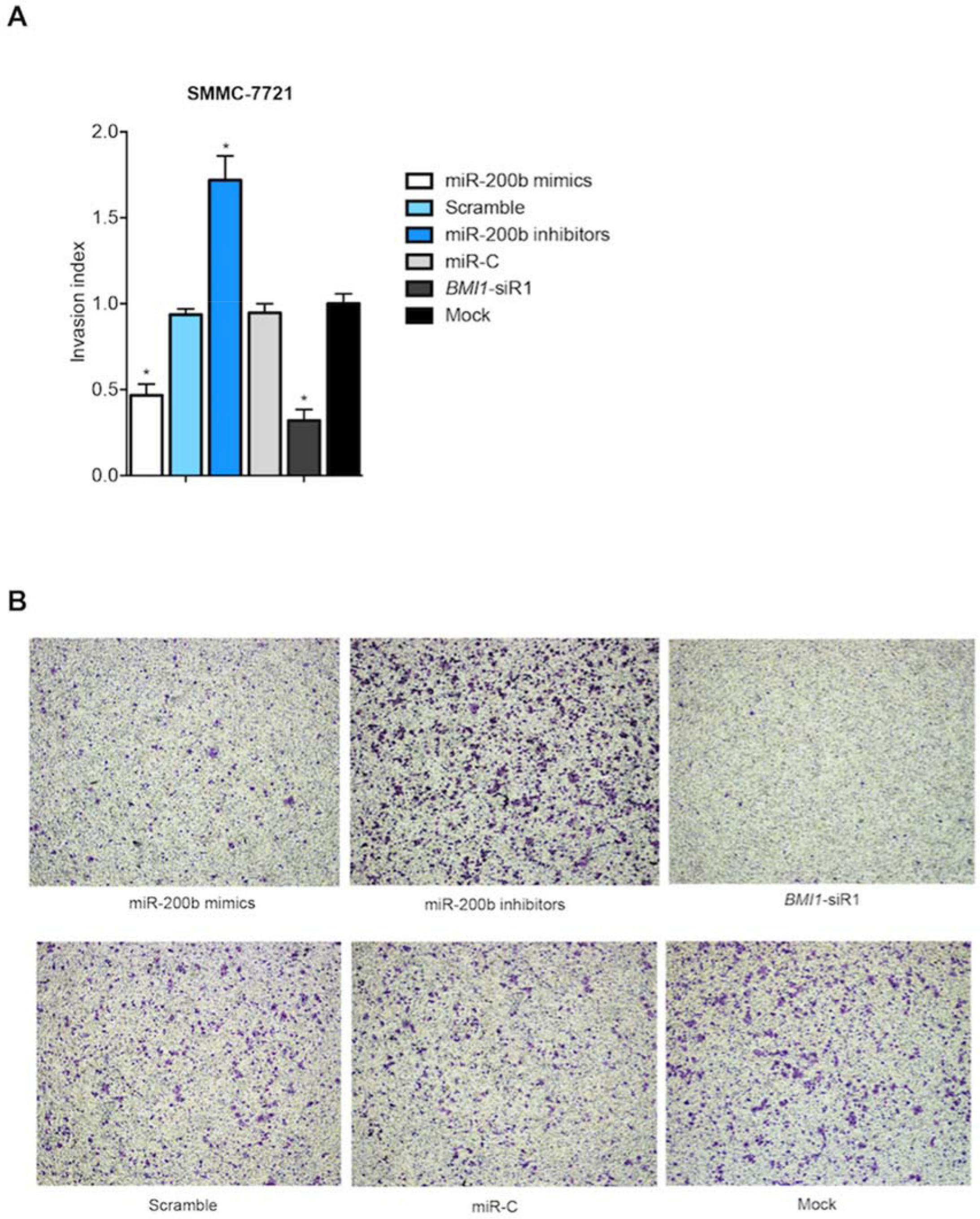

Figure S8: Transfection of miR-200b mimics or knockdown of *BMI1* inhibits invasion of SMMC-7721 cells. A. Transfection of miR-200b mimics or knockdown of *BMI1* inhibited invasion of SMMC-7721 cells. **B.** Representative results of miR-200b transfected or *BMI1* silenced SMMC-7721 cells that crossed the collagen-coated membranes within the invasion chambers. $*p<0.05$

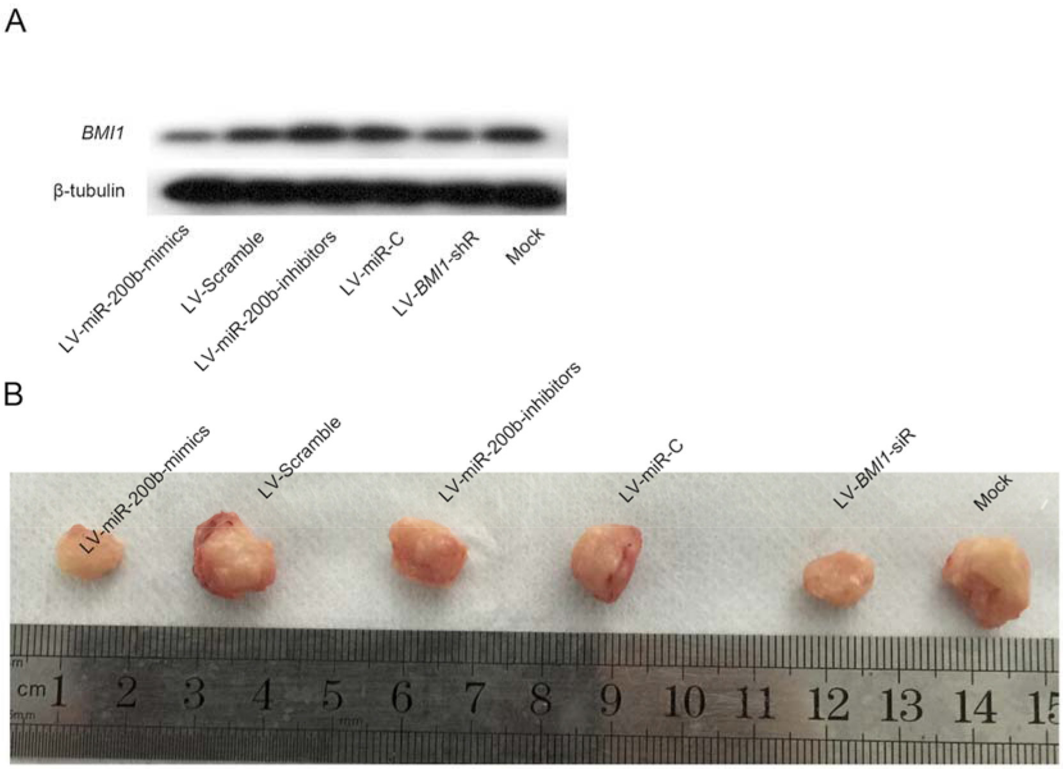

Figure S9: The effect of miR-200b/*BMI1* on tumor growth of HepG2 cells *in vivo*. A. Western blot results revealed that transfection of HepG2 cells with LV-miR-200b mimics, inhibitors and LV-*BMI1*-shRNA significantly modulated protein levels of *BMI1*. **B.** Representative results of LV-miR-200b-mimics or *BMI1*-shRNAtransfected cells.

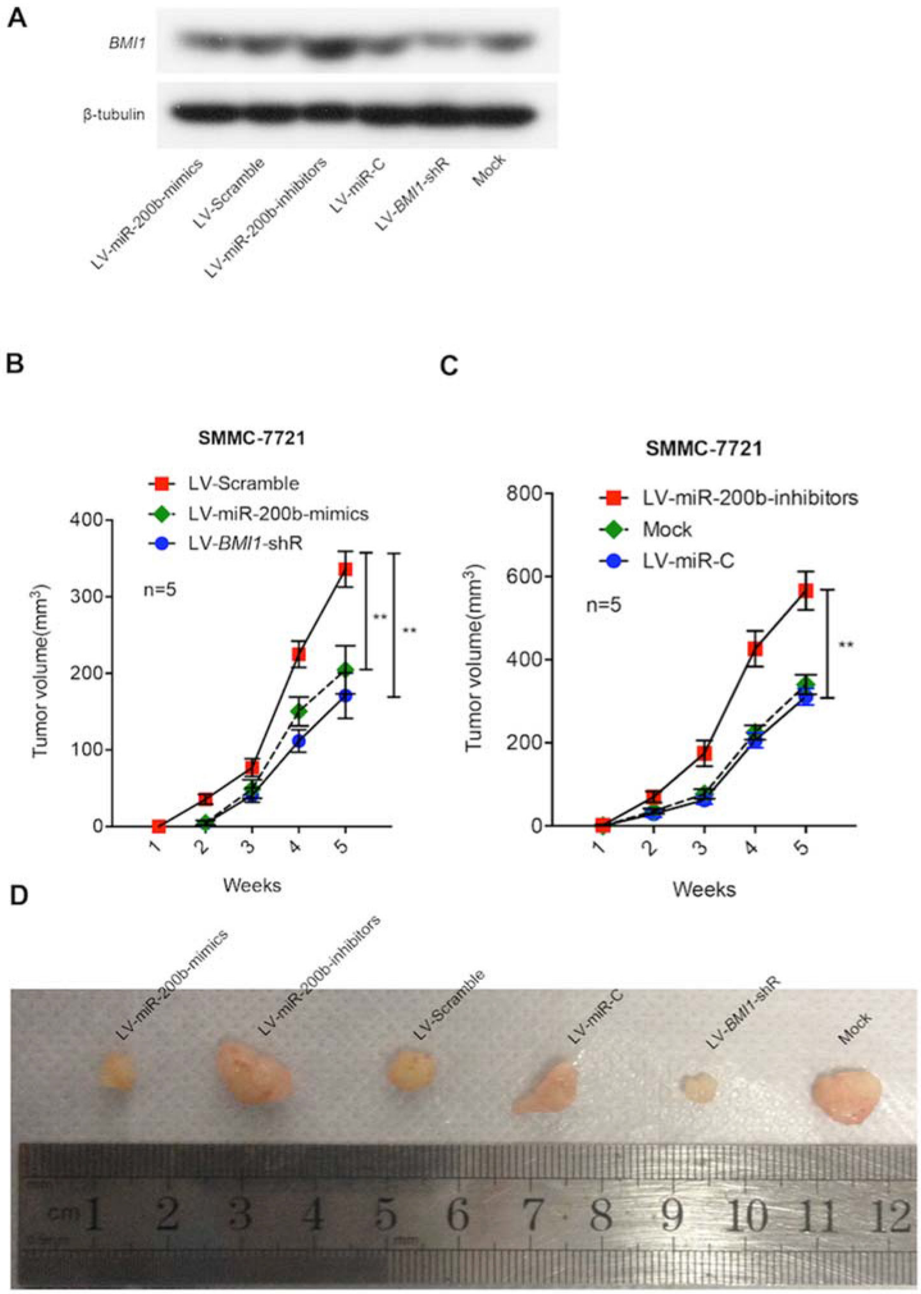

Figure S10: The effect of miR-200b/BMI1 on tumor growth of SMMC-7721 cells *in vivo*. A. Western blot results revealed that transfection of SMMC-7721 cells with LV-miR-200b mimics, inhibitors and LV-*BMI1*-shRNA significantly modulated protein levels of *BMI1*. **B, C** Tumor volume (n = 5) in mice inoculated with SMMC-7721 cells that stably-transfected with miR-200b mimics or *BMI1* shRNA. **D.** Representative results of LV-miR-200b-mimics or LV-*BMI1*-shRNA transfected cells.**p<0.01

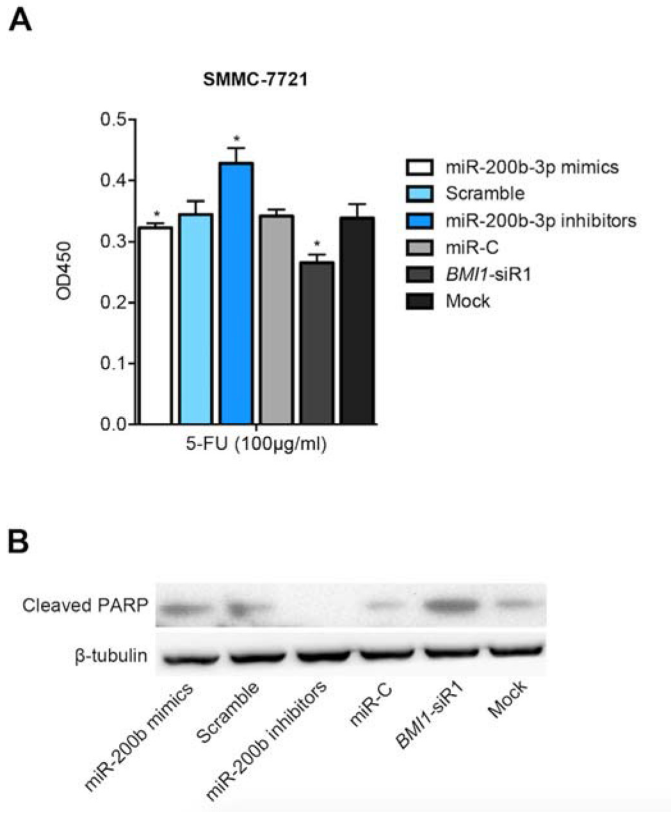

Figure S11: Transfection of miR-200b or knockdown of *BMI1* sensitizes SMMC-7721 cells to 5 FU-induced apoptosis. MiR-200b mimics/*BMI1*-siRNA reduced cell viability **A.** and increased the level of cleaved PARP **B.** of 5-FU-treated SMMC-7721 cells. *p <0.05

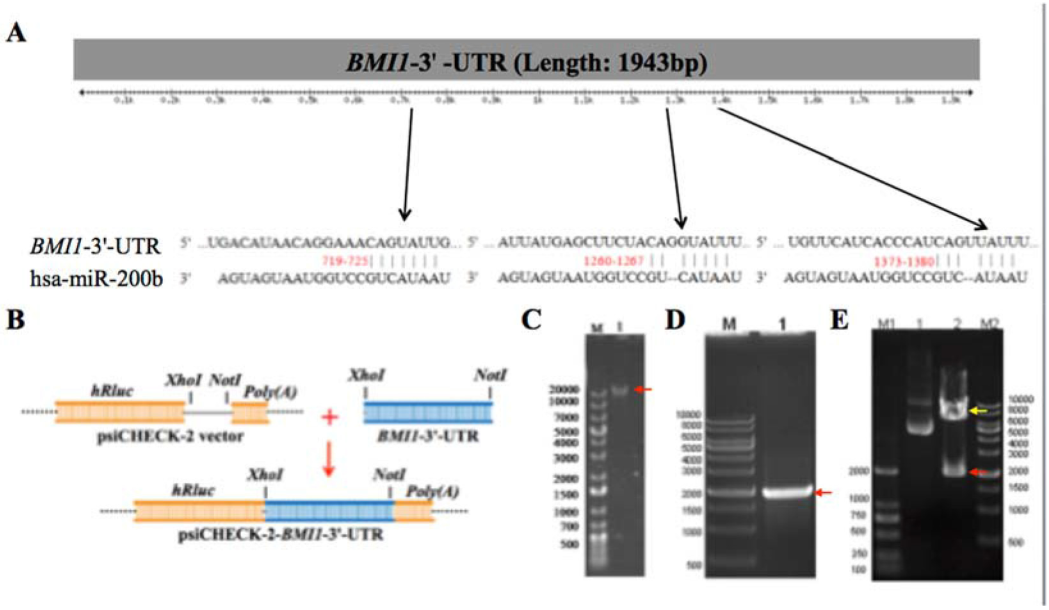

Figure S12: Diagram of psiCHECK-2-*BMI1*-3'-UTR vector construction. **A.** The potential miR-200b binding site in 3'-UTR of *BMI1*, as predicted by miRanda. **B.** *BMI1*-3'-UTR (wild type and mutant type) were cloned into a position downstream of luciferase. **C.** Identification of Human Genomic DNA. M: GeneRuler 1 kb plus DNA Ladder Marker; Lane 1: Human Genomic DNA. **D.** Identification of *BMI1*-3'-UTR. M: GeneRuler 1kb DNA Marker; Lane 1: *BMI1*-3'-UTR. **E.** Identification of psiCHECK-2-*BMI1*-3'-UTR vector. M1: GeneRuler DL2000 DNA Marker; Lane 1: psiCHECK-2-*BMI1*-3'-UTR vector; Lane 2: psiCHECK-2-*BMI1*-3'-UTR vector (after digestion; red arrow, *BMI1*-3'-UTR); M2: GeneRuler 1kb DNA Marker.

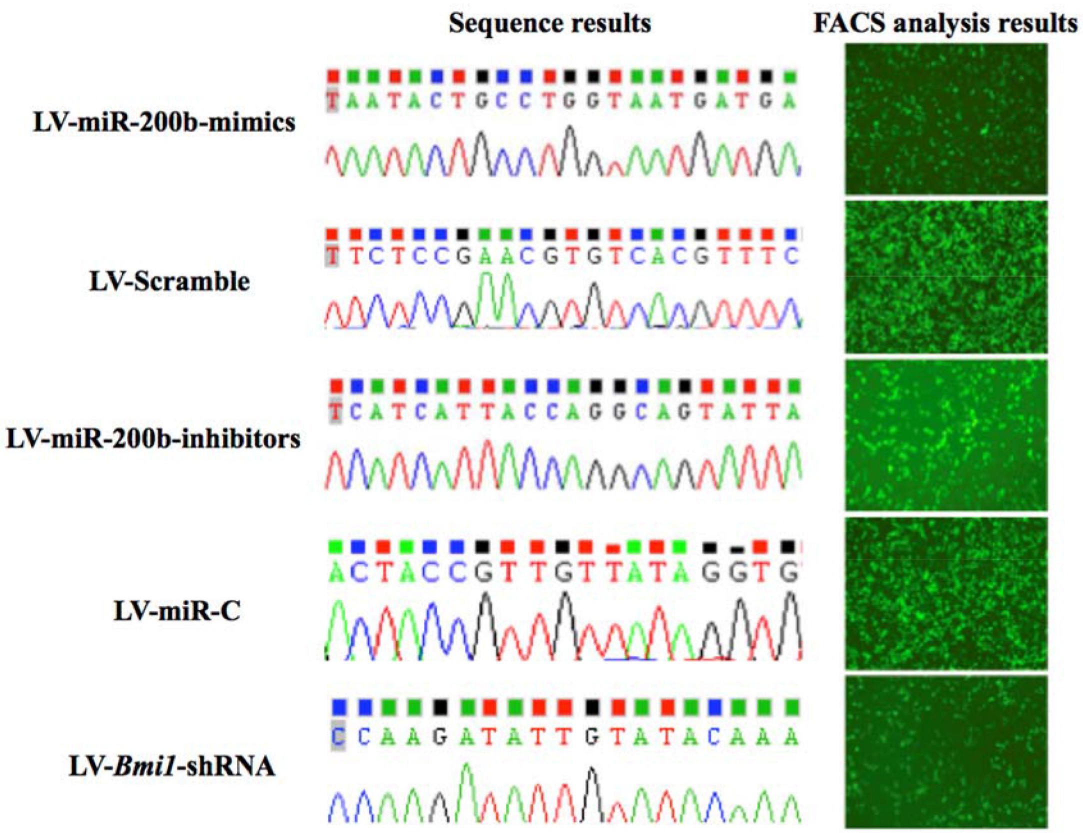

Figure S13: Generation of stably-transfected HCC cell lines for constitutive miR-200b and *BMI1*-shRNA expression.

Supplementary Table S1: Correlations of miR-200b and *BMI1* expression with clinicopathologic status in 36 cases of patients with HCC (Cohort 1)

Characteristics	N	miR-200b		P value	*BMI1*		P value
		Up	Down		Up	Down	
Age (years)				0.101			0.813
≤ 50	17	1	16		11	6	
> 50	19	5	14		13	6	
Sex				0.515			0.303
Male	34	6	28		22	12	
Female	2	0	2		2	0	
Hepatitis B virus antigen				0.506			0.293
Positive	26	5	21		16	10	
Negative	10	1	9		8	2	
α-fetoprotein				0.296			0.238
< 400 ng/mL	19	2	17		11	8	
≥ 400 ng/mL	17	4	13		13	4	
Tumor number				0.506			0.293
Single	26	5	21		16	10	
Multiple	10	1	9		8	2	
Tumor size				0.257			0.306
≤ 5cm	11	3	8		6	5	
> 5cm	25	3	22		18	7	
Capsular invasion				0.296			0.637
With	17	4	13		12	5	
Without	19	2	17		12	7	
Portal vein tumor thrombi				0.371			0.157
With	18	4	14		10	8	
Without	18	2	16		14	4	
Bile duct tumor thrombi				0.193			0.303
With	2	1	1		2	0	
Without	34	5	29		22	12	
Lymphatic metastasis				0.058			0.999
With	12	4	8		8	4	
Without	24	2	22		16	8	
Distant metastasis				0.999			0.303
With	2	0	2		2	0	
Without	34	6	28		22	12	
Tumor stage				515			0.999
I + II	9	1	8		6	3	
III + IV	27	5	22		18	9	
Histological grade				0.371			0.157
G1 + G2	18	4	14		10	8	
G3 + G4	18	2	16		14	4	

Supplementary Table S2: Clinicopathologic characteristics of patients (Cohort 2)

Characteristics	HCC (n=6)	Normal (n=6)
Age (mean ± standard deviation)	53.7 ± 9.6	51.2 ± 8.5
Hepatitis B surface antigen positive (yes/no)	4/2	1/5
Tumor status (T1/T2/T3/T4)	2/3/1/0	/
Lymph node status (N0/N1)	5/1	/
Distant metastasis (M0/M1)	6/0	/
TNM stage (I/II/III/IV)	2/3/1/0	/
Histological grade (G1/G2/G3)	2/3/1	/

Tumor stage and histological grade were classified in accordance with the criteria of American Joint Committee Cancer (AJCC) 7th Edition (2010).

Supplementary Table S3: Primer sequences used in the qPCR, MSP and BSP

Genename	Type	Sequence 5'-3'
miR-200b	Forward<br>Reverse	ACACTCCAGCTGGGTAATACTGCCTGGTAATG<br>CTCAACTGGTGTCGTGGA
miR-200b-BSP	Forward<br>Reverse	TATTTTTGGATTTTTGGAGGAGTT<br>ACCTAACTTATTAATTAACCTACCC
MSP- miR-200b - Unmethylated	Forward<br>Reverse	TGTTCTCTGTGGGGTGGGT<br>GGCTTAATCAATGGTGC
MSP- miR-200b - Methylated	Forward<br>Reverse	TGTTCTCTGTGGGGCGGGT<br>GGCTTAATCAATGGTGC
U6	Forward<br>Reverse	CTCGCTTCGGCAGCACA<br>AACGCTTCACGAATTTGCGT
BMI1	Forward<br>Reverse	ATCGGTTTAAACGCTACGCAAAACCGATCAAAG<br>CGATGCGGCCGCTGGGTATCTGCAAAGGTCGA
GAPDH	Forward<br>Reverse	ACAACTTTGGTATCGTGGAAG<br>ACAACTTTGGTATCGTGGAAG

Supplementary Table S4: MicroRNAs and small silencing RNAs used in the study

Name	Type	Sequence 5'-3'
miR-200b-mimics	Forward<br>Reverse	UAAUACUGCCUGGUAAUGAUGA<br>AUCAUUACCAGGCAGUAUUAU
miR-200b-inhibitors	Forward	UCAUCAUUACCAGGCAGUAUUA
Bmi-siRNA1	Forward<br>Reverse	CCAAGAUAUUGUAUACAAATT<br>UUUGUAUACAAUAUCUUGGTT
Bmi-siRNA2	Forward<br>Reverse	GACCUAAAUUUGUACAGUATT<br>UACUGUACAAAUUUAGGUCAA
Bmi-siRNA3	Forward<br>Reverse	GAAAAUAGCUAAGACUUUATT<br>UAAAGUCUUAGCUAUUUUCTA
Scramble	Forward<br>Reverse	UUUUCCGAACGUGUCACGUTT<br>ACGUGACACGUUCGGAGAATT
miR-C	Forward	GTTCTCCGAACGTGTCACGT

Supplementary Table S5: Primer sequences for amplification of *BMI1*-3'-UTR[1]

Gene name	Application	Type	Sequence (5'-3')
BMI1-3'-UTR	*BMI1*-3'-UTR-wt[2]	Forward	CCGCTCGAGTACCTGAGACTGTTAAGGAAAAAAATTTT
		Reverse	ATAAGAATGCGGCCGCTATTGGCAAACATTGGTAACTTTTACTT
	BMI1-3'-UTR-mut1[3]	Forward	TATTGGTATATGACATAACAGGAAAGTCATAAGTATGATATATTTATAAATGCTATA
		Reverse	TATAGCATTTATAAATATATCATACTTATGACTTTCCTGTTATGTCATATACCAATA
	BMI1-3'-UTR-mut2	Forward	AGAAGCTATTTATTATGAGCTTCTAGTCCATAATTTAAATAGAGCAAGCATGTTGAAT
		Reverse	ATTCAACATGCTTGCTCTATTTAAATTATGGACTAGAAGCTCATAATAAATAGCTTCT
	BMI1-3'-UTR-mut3	Forward	AACTTGGTGTGTGTTCATCACCCATGTCAATAATGTGAGGGTGTTTATTCTATATGAA
		Reverse	TTCATATAGAATAAACACCCTCACATTATTGACATGGGTGATGAACACACACCAAGTT

[1] GenBank accession number of *BMI1*: NM_005180.8

[2] The underlined parts of primers indicate the specific cleavage sites of restriction enzymes ("_", *XhoI*; "...", *NotI*).

[3] Potential target sequences of miR-200b according to miRanda miRNA target prediction algorithm are marked in red.

Supplementary Table S6: Oligonucleotides and primer sequences for generation of Lentiviral-miR-200b/*BMI1*-shRNA[1]

Name	Type	Sequence (5'-3')
hsa-miR-200b	Mature sequence	UAAUACUGCCUGGUAAUGAUGA
	With flanking sequences	CCAGCUCGGGCAGCCGUGGCCAUCUUACUGGGCAGCAUUGG<br>AUGGAGUCAGGUCUCUAAUACUGCCUGGUAAUGAUGACGGC<br>GGAGCCCUGCACG
miR-200b mimcs	Forward	GATCCGTAATACTGCCTGGTAATGATGATTCAAGAGATCATCAT<br>TCAGGCAGTATTACTTTTTTG
	Reverse	AATTCAAAAAAGTAATACTGCCTGAATGATGATCTCTTGAATCA<br>TCATTACCAGGCAGTATTACG
Scramble	Forward	GATCCGTTCTCCGAACGTGTCACGTTTCAAGAGAACGTGACAC<br>GTTCGGAGAACTTTTTTG
	Reverse	AATTCAAAAAAGTTCTCCGAACGTGTCACGTTCTCTTGAAACG<br>TGACACGTTCGGAGAACG
miR-200b inhibitors	Forward	GATCCGTCATCATTACCAGGCAGTATTATTCAAGAGATAATACT<br>GTGGTAATGATGACTTTTTTGAATTCAAAAAAGTCATCATTACC
	Reverse	ACAGTATTATCTCTTGAATAATACTGCCTGGTAATGATGACG
miR-C	Forward	GATCCGACTACCGTTGTTATAGGTGAGTTCAAGAGACTAGTGG<br>TTAAACATTTCACTTTTTTGAATTCAAAAAAGTGAAATGTTTAA
	Reverse	CCACTAGTCTCTTGAACTCACCTATAACAACGGTAGTCG
BMI1-shRNA	Forward	GATCCGCCAAGATATTGTATACAAATTCAAGAGATTTGTATACA<br>ATATCTTGGCTTTTTTG
	Reverse	AATTCAAAAAAGCCAAGATATTGTATACAAATCTCTTGAATTTG<br>TATACAATATCTTGGCG

[1] The underlined parts of primers indicate the specific cleavage sites of restriction enzymes ("____", *EcoRI*; "...", *BamHI*).
